# Effects of Microchannel Shape and Ultrasonic Mixing on Microfluidic Padlock Probe Rolling Circle Amplification (RCA) Reactions

**DOI:** 10.3390/mi9060272

**Published:** 2018-05-30

**Authors:** Yuri Ishigaki, Kae Sato

**Affiliations:** Department of Chemical and Biological Sciences, Faculty of Science, Japan Women’s University, Bunkyo, Tokyo 112-8681, Japan; kaesato@icl.t.u-tokyo.ac.jp

**Keywords:** microfluidics, padlock probe, rolling circle amplification, fluorescent hybridization

## Abstract

The fluorescence in situ hybridization (FISH)-based padlock probe and rolling circle amplification (RCA) method allows for the detection of point mutations. However, it requires multiple reaction steps and solution exchanges, making it costly, labor-intensive, and time-consuming. In this study, we aimed to improve the efficiency of padlock/RCA by determining the effects of microchannel shape and ultrasonic solution mixing. Using a circular-shaped microchamber and ultrasonic mixing, the efficiency of microfluidic padlock/RCA was improved, and the consumption of the expensive probe solution was reduced from 10 µL to approximately 3.5 µL. Moreover, the fluorescent probe hybridization time was reduced to 5 min, which is four times faster than that of the standard protocol. We used this method to successfully detect mitochondrial DNA and transcripts of β*-actin* and K-*ras* proto-oncogene codon 12 in cells. Our method offers improvements over current padlock/RCA methods and will be helpful in optimizing other microfluidics-based FISH-related analyses.

## 1. Introduction

In situ genetic analysis is a method of localizing and detecting specific DNA or mRNA sequences in morphologically preserved tissue sections or cell preparations by hybridizing a nucleotide probe to a complementary sequence of interest. The combination of a traditional morphological diagnostic method with a newer genetic methodology enables the identification of the genetic basis of human diseases, as well as individualized prevention strategies and early detection and treatment.

There are several in situ genetic analysis methods. Fluorescence in situ hybridization (FISH) was developed in the early 1980s [1] and has since become a well-known method for imaging genetic abnormalities. FISH methods have continued to develop over time, with substantial improvements to the signal-to-noise ratio from the development of RNAscope [2,3] and the Stellaris RNA FISH assay [4,5]. These methods are based on target signal amplification using a novel target probe design strategy and enable the simple detection of mRNA in cells. However, the detection of a point mutation or single-base substitution remains impossible using these methods.

In contrast, another FISH assay improvement, the in situ padlock probe and rolling circle amplification (RCA) method, allows for the detection of point mutations [6,7]. In this method, a target-specific padlock probe is circularized by enzymatic ligation to identify single-nucleotide sequence variants. Then, this circularized padlock probe is used as a template in a rolling circle replication reaction to amplify the specific signal for high assay sensitivity. This technique has been applied to the genotyping of single molecules of DNA or RNA in tissues and cells, including the identification of point mutations in mitochondrial DNA [8], the K-*ras* proto-oncogene [9,10], microRNAs [11], and infectious pathogens [12].

Compared to the standard FISH method, however, the multiple reaction steps and solution exchanges make padlock/RCA methods costly and labor-intensive. In addition, the current method requires long analysis times ranging from several hours to several days. Microfluidics enables the miniaturization, integration, automation, and parallelization of biochemical assays [13,14,15,16,17,18]. Thus, in order to overcome the limitations of current padlock/RCA methods, we previously developed a microfluidics-based padlock/RCA method in an attempt to achieve efficient reactions through the use of small reaction volumes and an automated system [19]. This automated microfluidics-based system performed the reaction solution exchange 13 times by means of a computer-controlled pump, reducing the number of labor-intensive manual operations. However, the system required 10 µL of reaction solution, which is more than the microfluidic chamber volume (2 µL), and the system was not optimized. Although there are several reports on microfluidics-based RCA [20,21,22], microfluidics-based in situ Padlock/RCA methods for cell imaging have not yet been reported, except for our method. In this study, we aimed to investigate the effects of the microchannel shape and ultrasonic solution mixing on padlock/RCA reaction efficiency. Several reports about mixers in microfluidics are available [23]. We chose ultrasonic mixing, because it is simple in configuration and easy to operate. These experiments were carried out to reduce the consumption of reagents and to improve target sequence and probe interaction.

## 2. Materials and Methods

### 2.1. Device Fabrication

Microfluidic devices were fabricated, as reported previously [19], with minor modifications. Briefly, a PDMS (Polydimethylsiloxane) sheet with an I-shaped microchannel pattern (width × depth × length, 1 mm × 200 μm × 10 mm) was fabricated by replica molding using a mold made with a PMMA (polymethyl methacrylate) sheet and a rectangular rod. A PDMS sheet with a circular-shaped microchamber pattern (4 mm in diameter) was fabricated by replica molding using a glass slide (76 × 26 mm; Matsunami, Osaka, Japan) and a circle punched from a PCR sealing film (4ti-0500; Nippon Genetics, Tokyo, Japan) using a 4-mm biopsy punch (Kai Industries, Seki, Japan). A PDMS pre-polymer solution (Silpot 184 W/C; Dow Corning Toray Co., Ltd., Tokyo, Japan) was poured into the mold to a thickness of 4 mm and cured in an oven for 1 h at 65 °C. The PDMS replica was peeled off, then placed on a glass slide and baked in an oven for 1 h at 100 °C. Through-holes were made with a 1.5-mm biopsy punch. The PDMS replica was bonded to a glass cover slip after plasma treatment. Wells were fabricated as reported previously [19] and consisted of a cover slip and a 2-mm thick PDMS sheet with a 5-mm diameter hole.

### 2.2. Device Cell Culture

Human epitheloid cervix carcinoma HeLa cells, human colorectal carcinoma HCT116 cells, and human choriocarcinoma BeWo cells were supplied by RIKEN BRC (Tsukuba, Japan). Cells were maintained in Dulbecco’s modified Eagle’s medium (DMEM) supplemented with 10% (HeLa and HCT116) or 15% (BeWo) fetal bovine serum (FBS; Thermo Fisher Scientific, Waltham, MA, USA) and 1× antibiotic-antimycotic (Thermo Fisher Scientific).

Cell suspensions were prepared at densities of 1.5 × 10^3^ cells/µL or 100 cells/µL and introduced into the microchannel (10 µL) or well (20 µL), respectively. The microchannel and well were manually precoated with 2 mg/mL Matrigel (BD Bioscience, San Jose, CA, USA) before introducing the cell suspension. The devices were humidified by wrapping them in a wet, lint-free wipe and plastic wrap. They were then incubated at 37 °C and 5% CO_2_ for 16 h to allow cells to adhere to the bottom of the device.

### 2.3. Padlock/RCA Reactions

Padlock/RCA reactions were performed manually with a micropipette. All reactions were performed under stopped-flow conditions. After each reaction, the reaction solution was removed with a micropipette, and a washing buffer was introduced into the device. After washing, the washing buffer was removed, and then the next reaction solution was introduced.

Detection of mitochondrial DNA was performed as follows. HeLa cells were fixed with 20 µL of 70% ethanol for 20 min, washed with 60 µL of Tris-acetate washing buffer (20 mM Tris-acetate, 10 mM magnesium acetate, 50 mM potassium acetate, 1 mM dithiothreitol, and 0.05% Tween-20, pH 7.9) at 23 °C, and then treated with 20 µL of 0.01% pepsin in 0.1 M HCl for 90 s at 37 °C, followed by washing with 60 µL of the washing buffer. In subsequent operations, the reaction solution volume was 3.5 µL for the microdevice and 20 µL for the well, with a washing buffer volume of 60 µL for every device. Target sequences were made accessible for hybridization by digestion with 0.5 U/µL *Msc*I restriction enzyme and 0.4 U/µL T7 exonuclease (both from New England Biolabs, Ipswich, MA, USA) at 37 °C for 40 min in 1× restriction enzyme buffer supplemented with 0.2 mg/mL bovine serum albumin (BSA; New England Biolabs). The microchannel was washed with the washing buffer at 25 °C.

Mitochondrial DNA-specific padlock probes were purchased from Sigma-Aldrich Japan (Hokkaido, Japan). A list of the probe sequences used for padlock/RCA in this study is shown in Table 1 [8]. Probe hybridization and ligation were performed in a single reaction with 100 nM probe and 0.1 U/µL T4 DNA ligase (Fermentas, Hanover, MD, USA) in 1× T4 ligase buffer with 0.2 mg/mL BSA and 250 mM NaCl at 37 °C for 30 min; the microchannel was then washed with washing buffer at 23 °C. The RCA reaction was performed with 1 U/µL Φ29 DNA polymerase (New England Biolabs), 0.25 mM dNTPs, 0.2 mg/mL BSA, and 5% glycerol in 1× phi29 polymerase buffer at 30 °C for 1.5 h. After polymerization, the microchannel was washed with washing buffer at 25 °C. The single-stranded RCA products (RCPs) were detected by hybridization with 250 nM Lin33 fluorescently labeled oligonucleotide probe (Eurofins Genomics, Tokyo, Japan) in a solution of 2× saline-sodium citrate and 8% formamide for 20 min at 37 °C. Cell nuclei were stained with 10 µg/mL Hoechst 33342 (Thermo Fisher Scientific) at 23 °C.

Detection of *KRAS* codon 12 and *ACTB* transcripts was performed as follows. A list of the probe sequences used for padlock/RCA in this study is shown in Table 1 [9,10]. BeWo and HCT116 cells in the circular microchamber were fixed with 20 µL of 3% PFA for 30 min and washed twice with 60 µL DEPC-PBS for 2 min each at 23 °C. The microchamber was dehydrated using a series of 70%, 85%, and 99.5% ethanol for 1 min each. The microchamber was then washed twice with PBS-T (DEPC-PBS and 0.05% Tween-20), and then treated with 20 µL of 0.1 M HCl for 10 min at 23 °C, followed by washing with 60 µL of PBS-T and M-MuLV RT Buffer (Promega, Madison, WI, USA). Then, 1 μM cDNA primer was added to the chamber with 20 U/μL of RevertAid H minus M-MuLV reverse transcriptase (Fermentas), 500 μM dNTPs, 0.2 mg/mL BSA, and 1 U/μL RiboLock RNase Inhibitor (Fermentas) in M-MuLV reaction buffer. This solution was incubated for 16 h at 37 °C. After incubation, the microchamber was washed briefly with PBS-T at 4 °C, followed by a post-fixation incubation for 30 min at 23 °C. After post-fixation, samples were washed with PBS-T and Ampligase DNA Ligase RXN Buffer (Epicentre, Madison, WI, USA). The next reaction was carried out with 100 nM of each padlock probe in a mix of 0.5 U/μL Ampligase (Epicentre), 0.4 U/μL RNase H (Fermentas), 1 U/μL RiboLock RNase Inhibitor, 50 mM KCl, and 20% formamide in Ampligase buffer. Incubation was performed first at 37 °C for 30 min, followed by 45 min at 45 °C. After ligation, the solution was washed with PBS-T and Φ29 DNA Polymerase Buffer at 4 °C. RCA was then performed with 1 U/μL Φ29 DNA polymerase in the supplied reaction buffer with 1 U/μL RiboLock RNase Inhibitor, 250 μM dNTPs, 0.2 mg/mL BSA, and 5% glycerol. Incubation was carried out for 2 h at 30 °C. After RCA, samples were washed with PBS-T, and RCPs were visualized using 100 nM of each corresponding detection probe in 2× SSC and 20% formamide at 37 °C for 15 min. The microchamber was then washed again with PBS-T and dehydrated using a series of 70%, 85%, and 99.5% ethanol for 30 s each. Cell nuclei were stained with 10 µg/mL Hoechst 33342 at 23 °C.

### 2.4. Ultrasonic Mixing

An ultrasonic toothbrush (AU-300D, ASAHI IRICA, Saitama, Japan), which vibrates at a frequency of 1.6 MHz with an intensity of 30 mW/cm^2^, was used as an ultrasonic apparatus. Because the temperature did not rise by the ultrasonic sonication with the low-power apparatus, thermal agitation seemed to be negligible. The brush head was removed from the handle, and the top of the handle without the brush head was pressed to the surface of the microdevice.

### 2.5. Image Analysis

Samples were visualized with an IX 71 fluorescence microscope (Olympus, Tokyo, Japan) with a PlanApo 60× oil immersion objective lens and Chroma 86009 BFP/GFP and DsRed filter set (380/450 nm and 555/620 nm excitation/emission for Hoechst 33342 and Alexa 555, respectively). Images were acquired with an EXi Blue camera (QImaging, Surrey, Canada) and processed using MetaMorph (Molecular Devices, Sunnyvale, CA, USA). RCPs were counted with ImageJ software (National Institutes of Health, Bethesda, MD, USA), and then the number of RCPs in an image was divided by the number of cells in the image. Finally, the mean of RCPs/cell values from 15 images was calculated.

## 3. Results and Discussion

### 3.1. Effect of Microchannel Shape

**Figure 1:**
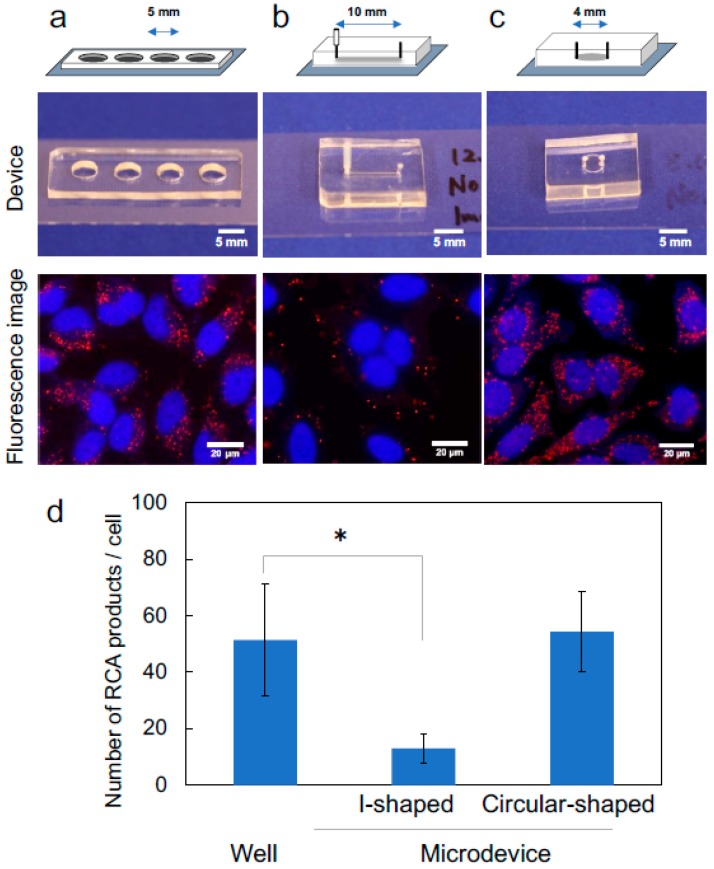
Effect of microchannel shape for detection of mitochondrial DNA. (**a**) Well for control experiments. (**b**) I-shaped microchannel device. (**c**) Circular-shaped microchamber device. Rolling circle amplification products (RCPs) from mitochondrial DNA in HeLa cells are visible in red; cell nuclei are in blue. The 2993-bp fragment was amplified using the padlock probe ppMSCs. (**d**) Numbers of detected RCPs per cell were digitally counted with ImageJ software. Data are shown as mean ± standard deviation (SD) of 15 images. * *p* < 0.05 (unpaired two-tailed Student’s *t* test).

We previously reported a microdevice-based padlock/RCA method with I-shaped microchannel [19]. The method required 10 µL of reaction solution, which is more than the microfluidic chamber volume (2 µL). This implies that shortage of the reagents may occur in the microchannel format resulting in poor reaction yields. We thought the shape of microchannels was an important parameter to achieve the desired performance. A well device (Figure 1a), I-shaped microchannel (the same as previously report [19], Figure 1b), and circular-shaped microchamber (Figure 1c) were used as reaction containers, and the number of RCPs detected in each device was compared. The results for the detection of mitochondrial DNA are shown in Figure 1d. Similar numbers of RCPs were observed in the well device and the circular-shaped microchamber. The volume and area of the bottom of each device are shown in Table 2. A reduction in reagent consumption (injection volume) from 10 µL (our previous result [19]) to 3.5 µL was achieved by using the circular microchannel.

In contrast, the I-shaped microchannel exhibited a lower reaction efficiency. Solution from the previous reaction or air bubbles sometimes remained in the corners of the I-shaped microchannel because of insufficient solution exchange. To investigate whether solution exchange was complete, the washing efficiencies of the I-shaped microchannel and circular-shaped microchamber were tested using fluorescein isothiocyanate (FITC)-dextran (40 kDa). Both microdevices were filled with 10^−5^ M FITC-dextran containing 0.05% Tween-20 and were washed with 10 µL PBS. FITC-dextran remained in both microdevices after a single washing step. However, when the devices were washed four times with PBS (10 µL each), FITC-dextran remained only in the I-shaped microchannel. In contrast, the circular-shaped microchamber had a low residual volume, and air bubbles were removed sufficiently; therefore, the microchamber was used in the subsequent experiments.

**Figure 2:**
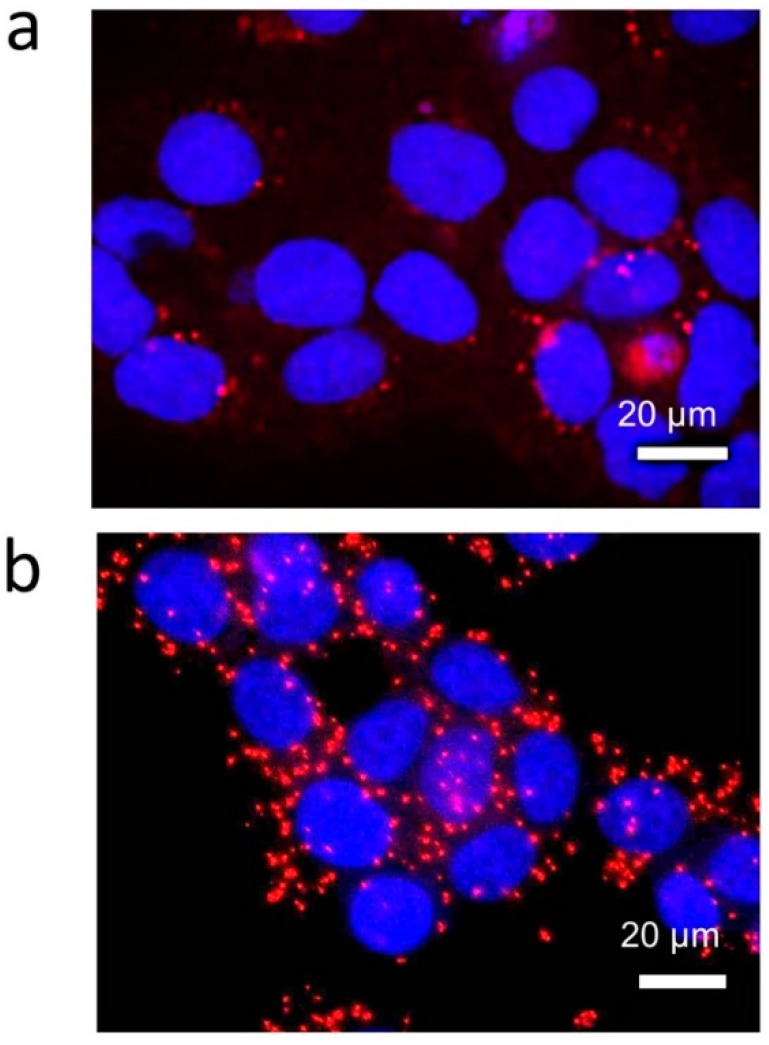
Imaging of mRNAs by rolling circle amplification (RCA) in the circular-shaped microchamber. (**a**) Detection of *KRAS* codon 12 (wild type) mRNA in the choriocarcinoma cell line BeWo. (**b**) Detection of β-actin mRNA in the colorectal carcinoma cell line HCT116.

The detection of *KRAS* codon 12 transcripts in BeWo cells and *ACTB* transcripts in HCT116 cells was performed using the circular-shaped microchamber. The number of RCPs derived from *ACTB* transcripts was larger than that derived from *KRAS* codon 12 transcripts (Figure 2a,b). The transcript levels of *ACTB*, which codes for a major structural protein β-actin, are known to be high, and our results are consistent with the previous knowledge.

### 3.2. Effects of Adsorption

Because microfluidic channels have larger surface area-to-volume ratios than wells, a portion of the enzymes in the reaction solution must be adsorbed onto the surfaces, making their reaction efficiencies low. Therefore, BSA was added to each reaction solution as an enzyme stabilizer [24] and adsorption inhibitor [25]. We assessed whether the BSA concentration affected the reaction efficiency. A concentration of BSA five times higher than that used in a previous report (0.2 mg/mL) [8] was used for mitochondrial DNA detection. The number of RCPs was 72.4 ± 20.1 per cell when using 1.0 mg/mL BSA and only 54.7 ± 10.9 per cell when using 0.2 mg/mL (*p* = 0.0056). Moreover, the fluorescence of RCPs was 1.3 times brighter when using 1.0 mg/mL BSA than when using 0.2 mg/mL BSA.

Next, we assessed whether the enzyme concentration decreased due to adsorption to the microchannel wall when using 1.0 mg/mL BSA. The microchannel was filled with an enzyme reaction solution for 5 min. Then, the solution collected from the microchannel was introduced into a new microchannel containing pretreated cells, and the reaction was performed. A reduction in the enzyme concentration would lead to a reduction in the reaction rate. Using the collected enzyme solution, 63.3 ± 13.2 RCPs/cell were detected, a count that was not significantly different from the control (57.3 ± 21.0 RCPs/cell, *p* = 0.356). Therefore, we concluded that a sufficient amount of enzyme was present for the microchannel reaction under the conditions used.

### 3.3. Effects of Ultrasonic Mixing

To improve the reaction efficiency of padlock/RCA, the probe, enzyme, and dNTPs must be transferred efficiently to their targets in the cell. It is assumed that the probe and enzyme molecules move by diffusion in the microchannel under static conditions. The time (*t*) required for the enzyme to move 2 mm (well depth) or 200 µm (microchannel depth) from the top of the solution to the cells on the bottom of each device was calculated using the Einstein–Smoluchowski Equation (1) [26]:

*x*^2^ = 2*Dt* (1)

where *x* is the distance and *D* is the diffusion coefficient.

The molecular weights of the padlock probe, T4 ligase, and Φ29 polymerase are approximately 30, 62, and 67 kDa, respectively. The diffusion constant of albumin (66 kDa) is *D* = 6.5 × 10^−7^ cm^2^/s [27], and thus the time required to move 2 mm or 200 µm was calculated to be 8.5 h or 5 min, respectively. Therefore, if the molecules moved only by diffusion, only a small portion of the enzyme molecules would be expected to take part in the reaction in the well within the reaction time frame. However, in the well, the reagents also move to their target by convection, allowing most of the reagents to take part in the reaction. In contrast, we expect no convection inside the microchannel because it is a small, enclosed space. Therefore, mixing of the solution in the microchannel is important for an efficient reaction, and therefore we tested the effect of ultrasonic mixing in the microchannel reactions.

**Figure 3:**
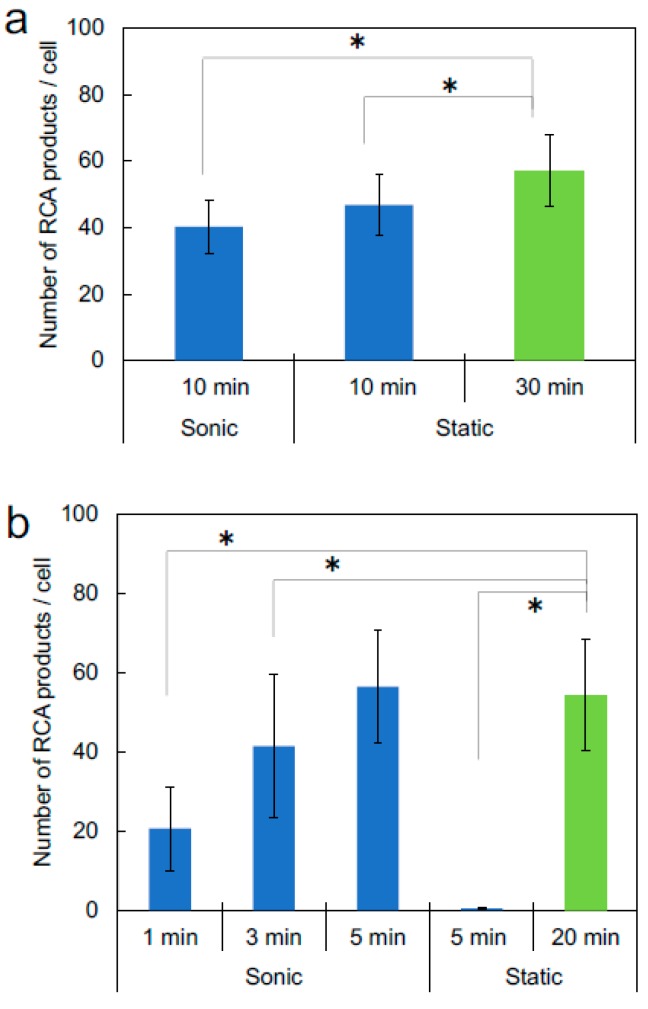
Effects of sonication on the number of detected RCPs. Target is mitochondrial DNA in HeLa cells. Green bars (control experiments) show results under the same reaction conditions used in a previous report [8]. Data are shown as mean ± SD (*n* = 15). * *p* < 0.05 (unpaired two-tailed Student’s *t* test). (**a**) Sonication during padlock probe hybridization and ligation step. (**b**) Sonication during the fluorescent probe hybridization step.

To assess whether ultrasonic mixing affects the reaction efficiency, ultrasonic mixing was performed during the padlock probe hybridization and ligation step or during the fluorescent probe hybridization step. The results for the detection of mitochondrial DNA are shown in Figure 3. In the padlock probe hybridization and ligation step, sonication did not affect the reaction efficiency (Figure 3a). The number of RCPs after the 10-min sonicated reaction was almost the same as that after the 10-min static reaction (40.3 ± 8.0 for sonic and 46.7 ± 9.2 for static RCPs/cell, *p* = 0.51). In contrast, during the fluorescent probe hybridization step, sonication increased the hybridization efficiency (Figure 3b). Although there were no RCPs generated after a 5-min static reaction, the number of RCPs generated by a 5-min sonicated reaction was similar to that achieved with a 20-min static reaction (56.5 ± 14.4 for sonic and 54.3 ± 14.1 for static RCPs/cell, *p* = 0.67). Thus, during the fluorescent probe hybridization step, it appears that solution mixing increased the supply of the fluorescent probe to the target RCPs, allowing for the observation of RCPs in a shorter time frame compared to that required under static conditions. Alternatively, sonication may have loosened aggregates of RCPs, increasing the hybridization of the fluorescent probe to the RCPs. In contrast, the sonication mixing had less of an effect on ligation efficiency because ligation is an enzymatic reaction-based process.

## 4. Conclusions

In conclusion, the circular-shaped microchamber exhibited improved performance for padlock/RCA reactions. Using ultrasonic mixing, the fluorescent probe hybridization time was reduced to 5 min, which is four times faster than that of the standard protocol [8,19]. Optimization of the microchannel shape and the hybridization conditions is a common challenge in other FISH-related analysis methods. Thus, our findings are not limited to padlock/RCA and will be helpful in optimizing other microfluidics-based FISH-related analyses.

## Figures and Tables

**Table 1:** Oligonucleotide sequences.

Name	Oligonucleotide Sequence (5′→3′)	Kind of DNA	Modification 5′	References
ppMscs	TAAGAAGAGGAATTGCCTTTCCTTTCCTACGACCTCAATGAACATGTTTGGCTCCTCTTCCCATGGGTATGTTGT	Padlock probe	Phosphate	[8]
Lin33Alexa555	CCTCAATGCACATGTTTGGCTCC	Detection probe	Alexa Fluor 555	[8]
P-KRAS	CC(L)TC(L)TA(L)TT(L)GT(L)TG(L)GA(L)TCATATTCGTC	cDNA primer	-	[9]
PLP-KRASwtGGT	GGCGTAGGCAAGAGTTCCTGTAGTAAAGTAGCCGTGACTATCGACTGAATCTAAGGTAGTTGGAGCTGGT	Padlock probe	Phosphate	[9]
DP-3	AGTAGCCGTGACTATCGACT	Detection probe	Cyanine3	[9]
P-ACTB	CG(L)GG(L)CG(L)GC(L)GG(L)ATCGGCAAAG	cDNA primer	-	[10]
PLP-b-actin_hum	GCCGGCTTCGCGGGCGACGATTCCTCTATGATTACTGACCTATGCGTCTATTTAGTGGAGCCTCTTCTTTACGGCGCCGGCATGTGCAAG	Padlock probe	Phosphate	[10]
DP-4	TGCGTCTATTTAGTGGAGCC	Detection probe	Cyanine3	[10]
(L) = Locked Nucleic Acid (LNA)-modified base				

**Table 2:** Volume and Bottom area of device.

Device	Size	Injection Volume (µL)	Chamber Volume (µL)	Bottom Area (cm^2^)	Volume Per Unit Area (µL/cm^2^)
Well device	5 mm diameter	20.0	20.0	20	1.0
I-shaped microchannel	width × depth × length, 1 mm × 200 μm × 10 mm	3.5	2.0	10	0.2
Circular-shaped microchamber	4 mm in diameter, 200 μm in depth	3.5	2.5	13	0.2
